# G3BP2, a stress granule assembly factor, is dispensable for spermatogenesis in mice

**DOI:** 10.7717/peerj.13532

**Published:** 2022-06-28

**Authors:** Damin Yun, Liwei Zhou, Jie Shi, Xinyao Li, Xiaolong Wu, Fei Sun

**Affiliations:** Nantong University, Institute of Reproductive Medicine, Nantong, Jiangsu, China

**Keywords:** G3bp2, Reproductive health, Male fertility, Spermatogenesis, CRISPR-Cas9, Apoptosis, Sperm, Testis, Conditional knockout, Germ cell

## Abstract

**Background:**

Spermatogenesis is a complex process that includes mitosis, meiosis, and spermiogenesis. During spermatogenesis, genetic factors play a vital role inthe formation of properly functioning sperm. GTPase-activating protein (SH3 domain)-binding protein 2 (G3BP2) is known to take part in immune responses, mRNA transport, and stress-granule assembly. However, its role in male fertility is unclear. Here, we generated a *G3bp2* conditional knockout (cKO) mouse model to explore the function of G3BP2 in male fertility.

**Methods:**

Polymerase chain reaction (PCR) and western blotting (WB) were used to confirm testis-specific *G3bp2* knockout. Hematoxylin-eosin (HE) staining to observe testicular morphology and epididymal structure. Computer-aided sperm analysis (CASA) to detect sperm concentration and motility. Terminal deoxynucleotidyl transferase-dUTP nick-end labeling (TUNEL) assay was used to detect apoptotic cells.

**Results:**

We found that cKO male mice are fertile with the normal morphology of the testis and sperm. Additionally, CASA of the semen from cKO mice showed that they all had a similar sperm concentration and motility. In addition, sperm from these mice exhibited a similar morphology. But the tunnel assay revealed increased apoptosis in their testes relative to the level in the wild type (WT).

**Conclusion:**

Together, our data demonstrate that G3BP2 is dispensable for spermatogenesis and male fertility in mice albeit with the increased germ-cell apoptosis.

## Introduction

Spermatogenesis is a precise and complex process that involves the self-renewal of spermatogonial stem cells and their differentiation into primary spermatocytes, which undergo two meiotic divisions to become haploid spermatids and then undergo a multi-phase maturation process, termed spermiogenesis, to form spermatozoa (de Kretser, Loveland & O.’Bryan, 2016; Fayomi & Orwig, 2018; Ibtisham & Honaramooz, 2020). The entire process is regulated by thousands of genes. Studies have shown that approximately 2,000 genes are expressed in the testis of the mouse (Roy Choudhury et al., 2010; Schultz, Hamra & Garbers, 2003). Among these genes, many, such as *Uhrf1*, *Ddx5*, *Zglp1*, *Pramef12*, and *1700102P08Rik* (Dong et al., 2019; Legrand et al., 2019; Nagaoka et al., 2020; Wang et al., 2019; Wu et al., 2020) are essential for spermatogenesis in the mouse, unlike others, such as *Golga4*, *Rai14*, and *Dnajb8* (Guo et al., 2020; Wang et al., 2020; Wu et al., 2021) are not essential. However, the functions of these spermatogenic genes, which are highly expressed in the testis, remain unclear. G3bp2 is one of the two GTPase-activating protein (SH3 domain)-binding proteins in the mouse. It is evolutionarily conserved in eutherians, involved in various biological processes, and highly expressed in the testis. Its core domain mainly consists of a nuclear transport factor two-like domain, SH3 domain-binding motifs, an arginine/glycine-rich box, and RNA-binding motifs (Gallouzi et al., 1998; Matsuki et al., 2013; Tourriere et al., 2001). As a core component, G3BP2 regulates the formation of stress granules through phase separation (Guillén-Boixet et al., 2020; Sanders et al., 2020; Yang et al., 2020). G3BP2 regulates breast-tumor initiation through the stabilization of Squamous cell carcinoma antigen recognized by T cells three (*SART3*) mRNA (Gupta et al., 2017). Additionally, it has been shown to interact with microRNA-23b to regulate fibrosis and proteinuria in diabetic nephropathy (Zhao et al., 2016). It is also involved in isoproterenol-induced cardiac hypertrophy through activating the NF-κB signaling pathway and plays an antiviral role by suppressing the viral replication upon viral infection of the cell (Eiermann et al., 2020; Hong et al., 2018; McCormick & Khaperskyy, 2017). However, a spermatogenic role has not been reported for this gene despite the high testicular expression. This study aimed to assess the function of G3BP2 in spermatogenesis. The Mouse Genome Informatics database states that knocking out this gene from the whole body leads to preweaning lethality in the mouse, and thus a conditional knock-out model has been generated (Bult et al., 2019). We observed that G3BP2 is mainly expressed in the cytoplasm of spermatogonia, spermatocytes, and round spermatids in the mouse. Secondly, *G3bp2*^loxp/loxp^ mating with stimulated by retinoic acid gene 8-Cre transgenic mice (*Stra8*-iCre), DEAD-box helicase 4-Cre transgenic mice (*Ddx4*-Cre) tool mice, and specifically knocked out this gene in germ cells at different stages (Fang et al., 2021; Sadate-Ngatchou et al., 2008). We observed that WT, *Stra8*-iCre; *G3bp2*^loxp/Δ^ (SKO), and *Ddx4*-Cre; *G3bp2*^loxp/Δ^ (DKO) mice were fertile, with normal testicular morphology, weight, and sperm concentration. Furthermore, no significant difference in testicular histology was observed among WT, SKO, and DKO mice. The stress granules also could form in heat-treated testis. However, the TUNEL assay revealed that both SKO and DKO mice displayed increased apoptosis compared with the level in WT mice. Collectively, our results indicate that G3BP2 is not essential for spermatogenesis and fertility in the mouse even though germ-cell–specific lack of this protein promotes germ-cell apoptosis.

## Materials and Methods

### Animals

The C57BL/6J background mice were purchased from the animal center of Nantong University. The animals are raised in the Laboratory Animal Center of Nantong University, where they are provided with free food and water under specific pathogen-free conditions with a 12 h/12 h light/dark cycle. All mice were treated humanely and killed by carbon dioxide asphyxiation to collect testicular and epididymis samples for further analysis. The Animal Ethical and Welfare Committee (AEWC) of Nantong University approved this research (Approval No. S20200630-502).

### Genotyping

Total DNA was extracted from the mouse tail by using the EasyPure Genomic DNA Kit (Transgen Biotech, Beijing, China), and the genotype was screened using PCR (EasyTaq DNA Polymerase, Transgen Biotech, Beijing, China). The primers are listed in Table 1.

**Table 1:** Primer sequences used in this study.

Gene name	Sequence (5′-3′)	Application
G3bp2-F	GTAAGCTGTAAGCCTGTGGAGT	Genotyping
G3bp2-R	GTAATAATAATCATGTCGAAAGAAAG	Genotyping
Stra8-iCre-F	AGATGCCAGGACATCAGGAACCTG	Genotyping
Stra8-iCre-R	ATCAGCCACACCAGACACAGAGATC	Genotyping
Ddx4-Cre-F	CACGTGCAGCCGTTTAAGCCGCGT	Genotyping
Ddx4-Cre-R	TTCCCATTCTAAACAACACCCTGAA	Genotyping
G3bp1-qF	AAGAACCTTCCTCCCAGTGG	RT-qPCR
G3bp1-qR	TCCACATCTCCTGGTTCACC	RT-qPCR
G3bp2-qF	CCCGAGTATTTGCACAGGTT	RT-qPCR
G3bp2-qR	TCACTCAAGGTTGCATGAGC	RT-qPCR
β actin-qF	AGCCATGTACGTAGCCATCC	RT-qPCR
β actin-qR	CTCTCAGCTGTGGTGGTGAA	RT-qPCR

### Generation of the *G3bp2* cKO mouse

Floxed *G3bp2* mice (*G3bp2*^loxp/loxp^) were generated from embryonic stem cells (ESCs) at Shanghai Research Center for Model Organisms. In brief, ESCs were injected with a mixture of donor (50 ng/µl), sgRNA1 (50 µg/ul), sgRNA2 (50 µg/ul), and Cas9 (100 µg/ul). The donor carried the sequence of *G3bp2* Exon3 flanked by loxP sites. The gene editor mice and are C57BL/6J background. *G3bp2*^loxp/loxp^ mice were confirmed *via* PCR. Mice with *Stra8*-Cre or *Ddx4*-Cre in the C57BL/6J background were purchased from the Jackson Laboratory. Stra8-Cre males were first crossed with *G3bp2*^loxp/loxp^ females to generate the *Stra8*-iCre; *G3bp2*^loxp/+^ male mice, then the *Stra8*-iCre; *G3bp2*^loxp/+^ male mice were crossed with *G3bp2*^loxp/loxp^ female mice to obtain the *Stra8*-iCre; *G3bp2*^loxp/Δ^ male mice (SKO). *Ddx4*-Cre; *G3bp2*^loxp/Δ^ male mice (DKO) were obtained likewise.

### Western blotting

Testicular extracts were prepared using RIPA lysis buffer (P0013C; Beyotime Biotechnology, Beijing, China) with the protease inhibitor PMSF (ST506; Beyotime Biotechnology, Beijing, China), and their protein content was resolved *via* 10% SDS-PAGE. Afterward, the protein bands were transferred from the gel onto a nitrocellulose membrane, which was then blocked with 5% nonfat milk in TBST for 1 h followed by overnight incubation with primary antibodies (Table 2) at 4 °C. Afterward, the membrane was washed three times with TBST and then incubated with secondary antibodies. The target protein bands were detected using an Amersham Typhoon 5 Biomolecular Imager (GE Healthcare Lifescience, Little Chalfont, UK).

**Table 2:** Antibodies used in this study.

Antibody name	Host species	Vendor	Catalog no	Working dilution
G3bp2	Rabbit	Proteintech	16276-1-AP	IF 1:200 IB 1:2,000
G3bp2	Rabbit	Abcam	Ab86135	IF 1:200 IB 1:2,000
TIA1	Rabbit	Proteintech	12133-2-AP	IF 1:200
PLZF	Mouse	R&D systems	MAB2944	IF 1:400
Beta Actin	Mouse	Proteintech	20536-1-AP	IB 1:3,000
GAPDH	Rabbit	Proteintech	10494-1-AP	IB 1:3,000
Mouse IgG (H+L)- Alexa Fluor 555	Goat	Thermo Fisher Scientific	A-21424	IF 1:300
Mouse IgG H&L- (IRDye® 680RD)	Goat	Abcam	ab216776	IB 1:3,000
Rabbit IgG H&L- (IRDye® 800CW)	Goat	Abcam	Ab216773	IB 1:3,000

### RNA isolation and RT-qPCR

All the organs (brain, heart, kidney, liver, lung, spleen, ovary, and testis) were collected from adult C57BL/6J background mice. Total RNA was extracted from these samples by using TRIzol reagent (Ambion, Foster City, CA, USA) and then reverse-transcribed to cDNA by using the PrimeScrip 1st Strand cDNA Synthesis Kit (TaKaRa, Beijing, China). Quantitative PCR was performed on a LightCycler 96 system (Roche, Mannheim, Baden-Württemberg, Germany) by using a TB Green Premix Ex Taq (Tli RNaseH Plus) Kit (TaKaRa) according to manufacturer protocol. The relative gene expression was quantified using the comparative cycle threshold method, with the β actin expression used for normalization. The primers are listed in Table 1.

### Hematoxylin-eosin (HE) staining

Mouse testis and epididymis were fixed in 4% paraformaldehyde at 4 °C for 24–48 h, embedded in paraffin wax, and sectioned at 5 μm. The sections were then deparaffinized and rehydrated as follows: Incubation in xylene for 20 min (performed twice), followed by successive incubations in 100% ethanol, 95% ethanol, 90% ethanol, 80% ethanol, 70% ethanol, and ddH_2_O for 2 min each. HE staining was carried out using the Hematoxylin-Eosin (HE) Staining Kit (E607318; Sangon Biotech, Shanghai, China).

### Immunofluorescence analysis

The testicular sections were prepared as described above for HE staining. Then antigen retrieval using the Improved Citrate Antigen Retrieval Solution (P0083; Beyotime Biotechnology, Beijing, China) was performed at 100 °C for 10 min. Subsequently, the samples were cooled and washed three times with 0.3% Triton X-100 in PBS for 10 min, followed by three PBS washes. Afterward, the samples were blocked with 1% BSA for 30 min and then incubated with primary antibodies (Table 2) in 1% BSA overnight at 4 °C. Subsequently, the samples were washed three times with PBS and then incubated with the secondary antibody for 1 h at room temperature. Next, the samples were washed three times with PBS, and DAPI (D9542) was used to stain the cell nuclei for 10 min at room temperature. Finally, the slides were mounted in glycerol (ZEISS, Oberkochen, Germany) and imaged using a ZEISS Axiocam 503 mono fluorescence microscope.

### Evaluation of sperm quality

Cauda epididymis was dissected from WT, SKO, and DKO male mice (10 weeks old, *n* = 5) and transferred to Tyrode’s solution (T1421-500 ml: Solarbio, Beijing, China). After incubating the samples at 37 °C for 10 min, sperm quality was measured using the CASA system of the Hamilton Thorne CEROS II (Medealab™, Erlangen, Germany).

### TUNEL assay

Testes were fixed in 4% paraformaldehyde, embedded in paraffin, and sectioned (5 µm). The sections were then deparaffinized and rehydrated as described above, and then incubated with protease K (20 µg/ml) at room temperature for 30 min to the TUNEL assay was performed using the One-Step TUNEL Apoptosis Assay Kit (C1089; Beyotime, Beijing, China) by following the instructions of the manufacturer, and then the nuclei were stained with DAPI. Images were obtained using a ZEISS Axiocam 503 mono fluorescence microscope. We repeated the experiment five times.

### Fecundity test

Two female mice (at age of 8 weeks) were caged with each test male mouse (WT, SKO, and DKO, at age of 8 to10 weeks), and copulation was evaluated by checking each morning for a vaginal plug. The number of pups was counted on the day of birth.

### Heat stress assay

Adult (10 weeks) male mice (WT, SKO, and DKO) were used for the heat-treatment experiment (Kim, Cooke & Rhee, 2012). Mice were anesthetized and the lower third of the body was placed in the water bath at 42 °C for 20 min. The mice were sacrificed after heat treatment and the testis were collected for further analysis.

### Statistical analysis

All data were reported as mean ± SD from at least three replicates for each experiment. Significance testing of student T-tests using Prism 7.0 software using two-tailed failure. *P* < 0.05 was considered statistically significant. NS means not significant.

## Results

### *G3bp2* is highly expressed in the mouse testis

**Figure 1:**
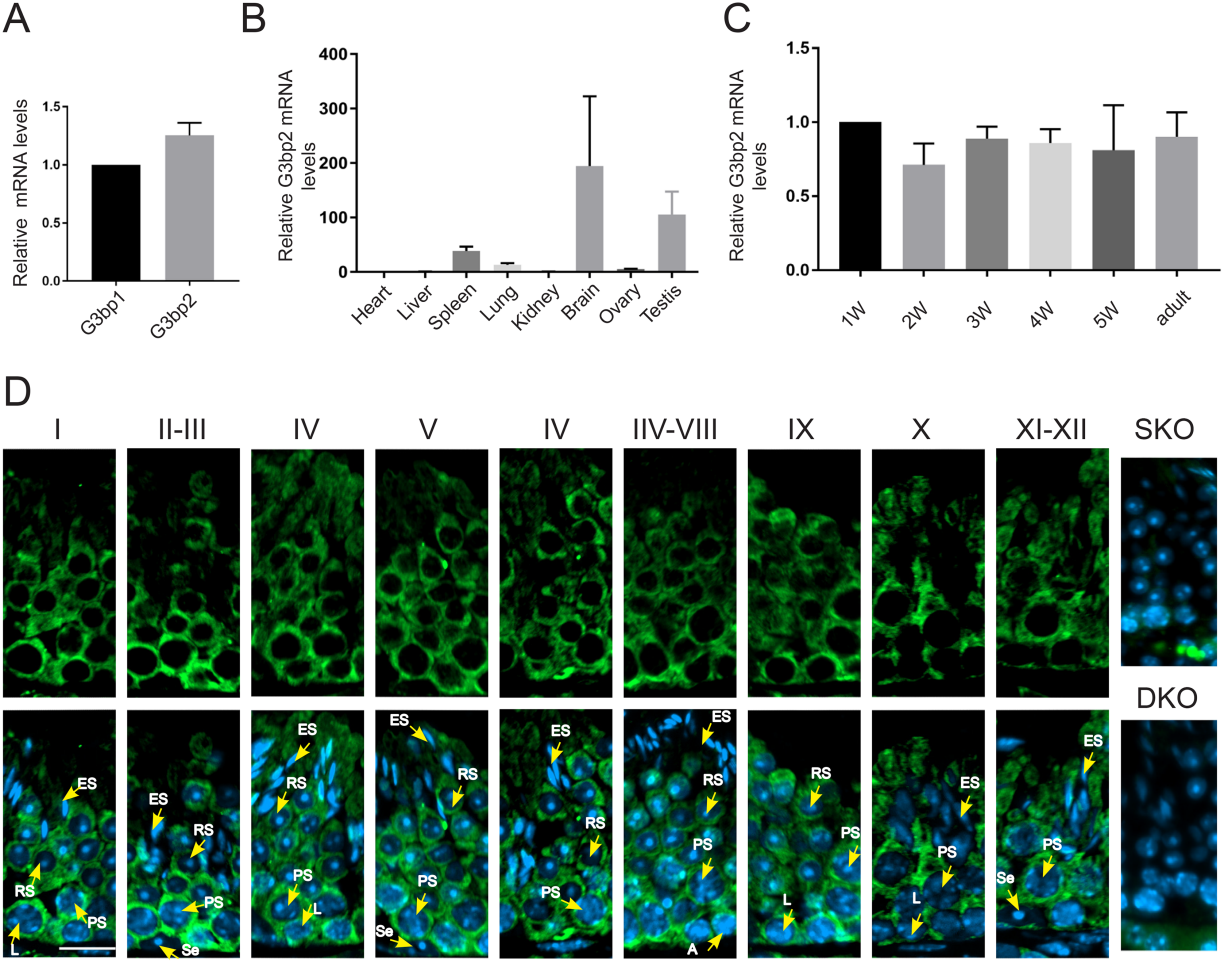
*G3bp2* highly expressed in mouse testis. (A) RT-qPCR analyses of *G3bp1/2* mRNA expression levels in adult mouse testis. Beta-actin as an internal positive control. (B) RT-qPCR analyses of *G3bp2* mRNA expression levels in different organs. Beta-actin as an internal positive control. Data are presented as mean ± SD, *n* = 3. (C) RT-qPCR shows the G3BP2 mRNA levels at different ages in wild-type mice. Beta-actin is the internal positive control. (D) Immunofluorescent staining of G3BP2 (green) in testicular sections from wild-type, SKO, and DKO mouse. Nuclei were stained with DAPI. The stages of the cycle of the seminiferous epithelium were characterized by I–XII. Se, Sertoli cell; A, A-type spermatogonia; PS, Pachytene spermatocyte; L, leptotene spermatocyte; RS, round spermatids; ES, elongated spermatids. Each is marked with a yellow arrow. Scale bar = 20 μm. Antibody from Proteintech (16276-1-AP).

G3bp family containing G3bp1 and G3bp2, G3bp2 is high expression than G3bp1 in adult testis by reverse transcription-quantitative polymerase chain reaction (RT-qPCR) (Fig. 1A). To explore the spatial expression pattern of *G3bp2*, we used RT-qPCR and observed high expression in the spleen, brain, and testis (Fig. 1B). Next, we assessed the temporal expression pattern of *G3bp2*. Toward this end, the testes from adult mice and those aged 1–5 weeks were collected. RT-qPCR analysis showed G3BP2 expression in mouse testis at all stages (Fig. 1C). To evaluate the cellular expression and subcellular localization patterns, we performed immunofluorescence analysis of testis sections for G3BP2. G3BP2 was mainly detected in the cytoplasm of spermatogonia, spermatocytes, and round spermatids, and not detected signals in SKO and DKO (Fig. 1D).

### *G3bp2* conditional knockout (cKO) mice displayed normal spermatogenesis and fertility

**Figure 2:**
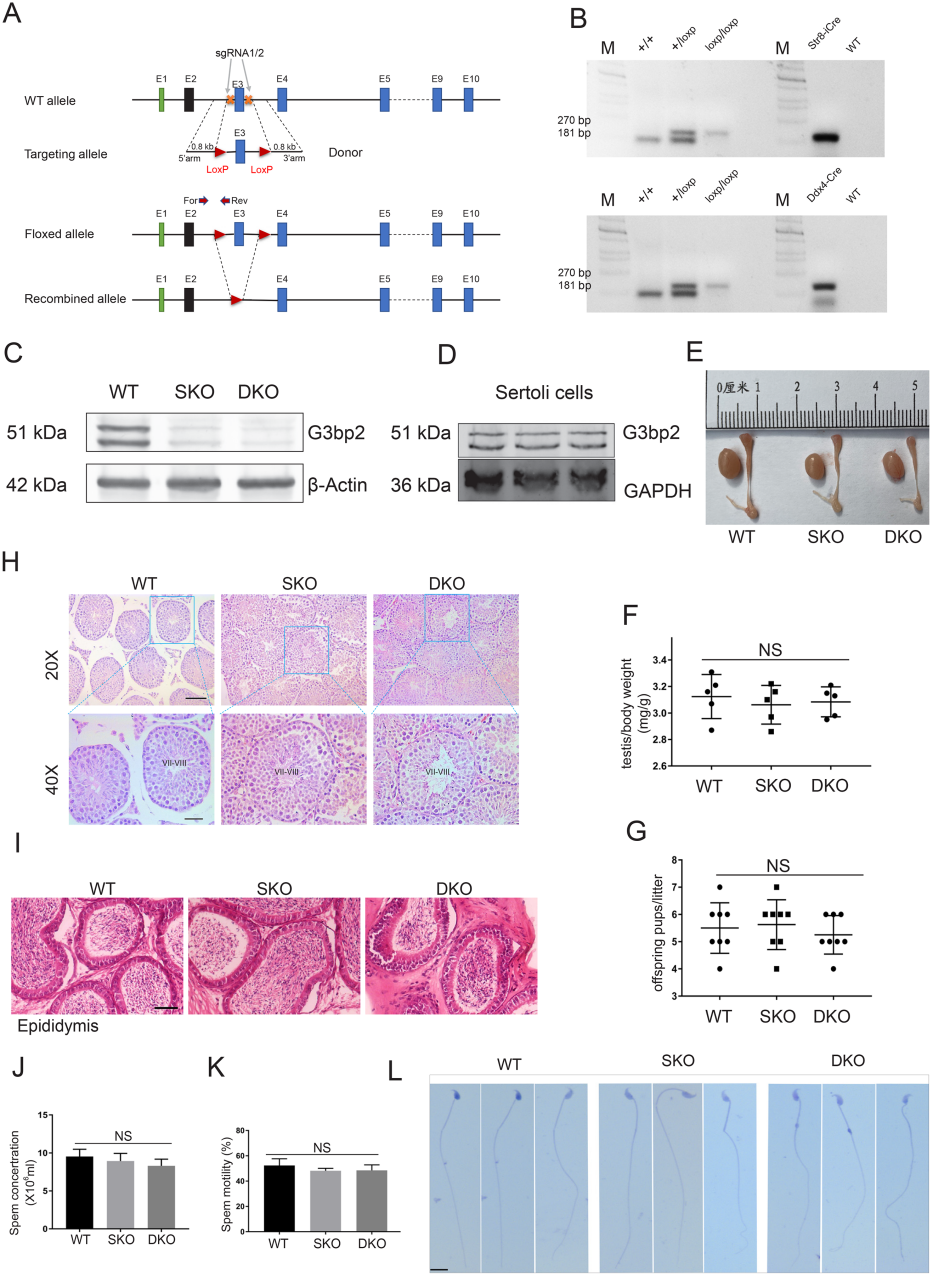
*G3bp2* cKO mouse displayed normal spermatogenesis and fertility. (A) Schematic representation of the targeting strategy for generating a floxed *G3bp2* allele through the CRISPR/Cas9 system in murine embryonic stem cells. Exon 3 will be deleted after Cre-mediated recombination. The G3BP2 protein begins Exon 1, which would cause frameshift mutation when Exon 3 deletion. The position of the forward (For) and reverse (Rev) primers used for genotyping are shown. (B) Genotyping results showing the floxed (loxp) and the WT (+) alleles can be detected at 181 and 270 bp bands, respectively. (C) Western blotting shows the G3BP2 protein levels in WT, SKO, and DKO adult testis. **β** actin as a loading control. Antibody from Abcam (Ab86135). (D) Immunoblotting shows the G3bp2 low expression in isolated Sertoli cells. GAPDH as a loading control. Antibody from Abcam (Ab86135). (E) The morphology of WT, SKO, and DKO adult mouse testis and epididymis. (F) The testis body weight ratio between WT, SKO, and DKO. Data are presented as mean ± SD, *n* = 5. (G) The fertility test and offspring pups form WT, SKO, and DKO. Data are presented as mean ± SD, *n* = 5–8. (H, I) Hematoxylin and Eosin Staining of testis and epididymis from WT, SKO, and DKO Adult mice. Scale bar = 50 μm, 40X = 20 μm. (J, K) Sperm count and motility of the cauda epididymal from WT, SKO, and DKO adult mice. Data are presented as mean ± SD, *n* = 5. (L) Sperm morphology from WT, SKO, and DKO mice by H&E staining at 10-week-old. Scale bar = 20 μm. NS, no significant.

Because whole-body *G3bp2* knockout in the mouse leads to preweaning lethality (Bult et al., 2019), we generated germline-specific *G3bp2* knockout mice. Toward this end, we inserted a loxP site at each end of exon 3 of the *G3bp2* gene (*G3bp2*^loxp/loxp^) (Fig. 2A). To explore the effect of germ-cell–specific *G3bp2* knockout on spermatogenesis during different stages of post-natal development, we used *stra8*-iCre mice, which begin expressing Cre recombinase in germ cells at 3 days post-partum (dpp) (Sadate-Ngatchou et al., 2008), and *Ddx4*-Cre mice, which begin expressing Cre recombinase in germ cells at embryonic stage 15 days (Gallardo et al., 2007). *G3bp2*^loxp/loxp^ mice were crossed with these mice to generate the following two types of germline-specific *G3bp2* knockout mice: *Stra8-*iCre; *G3bp2*^loxp/Δ^ (SKO), *Ddx4-*Cre; *G3bp2*^loxp/Δ^ (DKO). PCR was used to confirm testis-specific *G3bp2* knockout, immunostaining, and western blotting were used to confirm the KO efficiency (Figs. 2B, 2C and 1C). There is still a very faint G3bp2 signal in KO mouse testis, which is presumably Sertoli cell protein. Furthermore, we detect a weak bands by isolate Sertoli cells protein (Fig. 2D). However, sertoli-cell–specific deletion of *G3bp2* by using Amh-Cre tool mice did not impact male fertility (data not shown).

We observed that there was no significant difference in testicular morphology among wild-type (WT), SKO, and DKO mice of the same week of age (Fig. 2E). The testis body weight ratio and the fertility test showed no difference in litter size among WT, SKO, and DKO mice (Figs. 2F and 2G). Moreover, full spermatogenesis and normal epididymal structure were observed in SKO and DKO mice in comparison with the WT mice (Figs. 2H and 2I). Additionally, computer-aided sperm analysis (CASA) of the semen from WT, SKO, and DKO mice showed that they all had a similar sperm concentration and motility at 10 weeks old (Figs. 2J and 2K). In addition, sperm from these mice exhibited a similar morphology (Fig. 2L). These results indicate that G3BP2 is not essential for spermatogenesis and male fertility in the mouse.

### G3BP2 deficiency-induced germ-cell apoptosis

**Figure 3:**
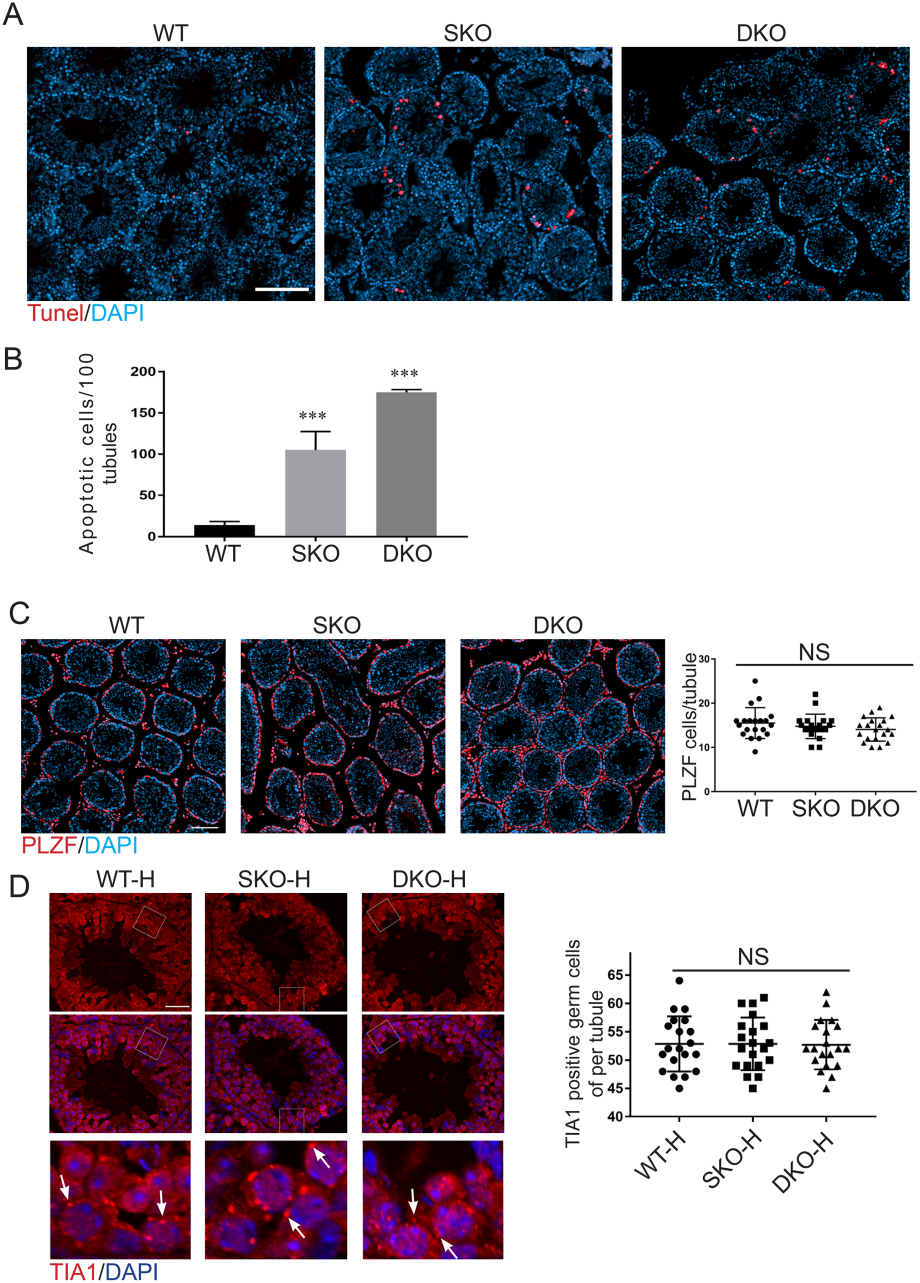
G3BP2 deficiency-induced germ cell apoptosis. (A) Cell apoptosis detected by TUNEL assay from WT, SKO, and DKO adult mice. Apoptotic cells are marked red and nuclei were stained DAPI, scale bar = 100 μm. (B) Tunel positive cells in 100 tubules at WT, SKO, and DKO adult mice. Data are presented as mean ± S D, *n* = 5. ***, *p* < 0.001 by student’s t-test. (C) Immunodetection of PLZF in testis sections from adult WT, SKO, and DKO mice. Scale bar: 50 μm. Quantification of PLZF-positive cells in seminiferous tubule from WT, SKO, and DKO mice. A total of 20 tubules were counted. (D) Heat stress adult testis sections stained for TIA1, a marker of stress granules. Magnified images are shown at the bottom. The condensed stress granules are marked by white arrow. Scale bar: 20 μm. Quantification of TIA1-positive cells in seminiferous tubule from WT, SKO, and DKO mice. A total of 20 tubules were counted. NS, no significant.

The TUNEL assay is used to detect the DNA breaks during apoptosis. To have a deeper understanding of the influence of *G3bp2* deficiency on reproduction, we performed the TUNEL assay on testicular sections from WT, SKO, and DKO mice at 10 weeks old. Surprisingly, SKO and DKO displayed more apoptotic germ cells than in WT testis (Figs. 3A and 3B), indicating that G3BP2 deficiency promotes germ-cell apoptosis. There are high levels of germ cell death in the G3BP2 KO testis, but no significant difference in sperm concentration. So we speculate there is higher spermatogonia proliferation to balance the loss of germ cells. The WT, SKO, and DKO mouse testis sections were staining PLZF, a spermatogonia marker (Song et al., 2020). The seminiferous tubules from WT, SKO, and DKO contained similar numbers of spermatogonia cells (Fig. 3C). Stress granules condense into being through liquid-liquid phase separation (LLPS), G3bp1 and G3bp2 are the core regulatory components of stress granules, so we speculated whether elimination of G3bp2 has any effect on the formation of stress granules in testis (Guillén-Boixet et al., 2020; Yang et al., 2020). WT, SKO, and DKO mice were killed immediately after being heated in a 42 °C water bath for 20 min and the heat stress testis section was staining TIA1, a stress granules marker (Kim, Cooke & Rhee, 2012; Mackenzie et al., 2017). Statistical analysis of TIA1 positive cells after heat stress, there was no significant difference in WT, SKO, and DKO, suggesting that G3bp2 deletion in not affect stress granules assembly (Fig. 3D).

## Discussion

Spermatogenesis is a precise and complex process involving spatiotemporal regulation of gene expression. Numerous genes have been demonstrated to play important roles in spermatogenesis. However, there are still many genes with high testicular expression whose functions remain unknown (Dong et al., 2019; Schultz, Hamra & Garbers, 2003; Nagaoka et al., 2020). The current study revealed that G3BP2 is dispensable for spermatogenesis and male fertility even though G3BP2 deficiency increases germ-cell apoptosis. G3BP2 was found mainly in the cytoplasm of spermatogonia, spermatocytes, and round spermatids. Despite this broad expression pattern in the male germline, germ-cell–specific deletion of *G3bp2* did not affect spermatogenesis. There is still a very faint G3bp2 signal in KO mouse testis, which is presumably Sertoli cell protein. We observed a low-level expression of *G3bp2* in Sertoli cells *via* western blotting, and Sertoli-cell–specific deletion of *G3bp2* by using Amh-Cre tool mice did not impact male fertility, either (data not shown). G3BP1 and G3BP2 proteins are 59% identical, and the protein domain displays an identical overall molecular organization (Prigent et al., 2000). Thus, there might be redundancy between the functions of G3BP1 and G3BP2, and G3BP1 may have compensated for the loss of G3BP2 in the cKO mouse. Although the KO strategy resulted in G3bp2 frameshift mutations, the truncated protein remains (containing Exon 1 and 2), whose function needs further validation. *G3bp2* is mainly expressed in the mouse brain and testis. The mouse brain is the center of the nervous system, playing vital coordination. Mouse testis is the male gonad, whose function consists of the production of sperm and synthesis/secretion of hormones. However, several pieces of evidence indicate marked cellular and molecular similarities between the brain and testis, including a similar gene expression pattern (Matos et al., 2021). Although *G3bp2* deficiency increased germ-cell apoptosis, this gene was not found essential for spermatogenesis. Our data would provide a basic experiment on the function of G3BP2 in the brain.

Stress granules (SGs) are membrane-free ribonucleoprotein-based cytoplasmic compartments that form when cells are exposed to stress, such as heat, hypoxia, or oxidative stress (Hofmann et al., 2021). G3BP2 is a core component of SGs, and whether its deficiency affects SG formation is unknown. It has been reported that knocking out the *Mage-b4* and *Mage-b10* gene or overexpression of G3BP1 makes the male germline hypersensitive to heat stress *in vivo* (Lee et al., 2020). Our study revealed that germ-cell–specific lack of *G3bp2* does not affect spermatogenesis under normal conditions, and, even though the elimination of G3bp2 does not affect the formation of stress granules in heat-treated testis.

The results from the TUNEL assay showed that SKO and DKO mice had more apoptotic spermatogenic cells than WT mice, and the apoptotic signals were mostly in spermatogonia and spermatocytes. Thus, we speculate that G3BP proteins may play a role in germ-cell survival in male mice. G3bp2 is a core component of stress granules, which protect germ cells from heat stress (Huelgas-Morales et al., 2016; Kim, Cooke & Rhee, 2012; Sanders et al., 2020). Thus, without G3BP2, germ cells may be more sensitive to the changes in the environment and predisposed to apoptosis. Meanwhile, spermatogenesis is a process that undergoes both numerical and morphological changes, and the lack of G3bp2 may affect cell autoregulation (de Kretser, Loveland & O.’Bryan, 2016). The previous study had shown that approximately 70% of spermatogonia are estimated to undergo apoptosis under the physiological condition in spermatogenesis (de Kretser et al., 1998; Ogawa, Ohmura & Ohbo, 2005). Although increased apoptosis in germ cells of cKO testis, the remained germ cells could produce enough sperm, so the fertile phenotype is almost unaffected. Accordingly, we plan to explore the causative factors for the apoptotic signals in the cKO mice.

In summary, we generated two male-germline–specific *G3bp2* knockout mice (*Stra8-*iCre; *G3bp2*^loxp/Δ^ and *Ddx4-*Cre; *G3bp2*^loxp/Δ^), which showed no difference in spermatogenesis or semen parameters from WT mice. However, both of these transgenic lines showed increased apoptosis in the male germline compared with the WT level. Our data provide valuable information for male reproductive health.

## Supplemental Information

10.7717/peerj.13532/supp-1 Supplemental Information 1 ARRIVE Checklist.

10.7717/peerj.13532/supp-2 Supplemental Information 2 Apoptotic cells100 tubules.

10.7717/peerj.13532/supp-3 Supplemental Information 3 G3bp2_week_expression.

10.7717/peerj.13532/supp-4 Supplemental Information 4 offspring per litter.

10.7717/peerj.13532/supp-5 Supplemental Information 5 Sperm motility.

10.7717/peerj.13532/supp-6 Supplemental Information 6 G3bp2 SKO IF.

10.7717/peerj.13532/supp-7 Supplemental Information 7 Testis body weight ratio.

10.7717/peerj.13532/supp-8 Supplemental Information 8 G3bp2 DKO IF.

10.7717/peerj.13532/supp-9 Supplemental Information 9 G3bp2_expression_in_sertoli_cell.

10.7717/peerj.13532/supp-10 Supplemental Information 10 Organism expression.

10.7717/peerj.13532/supp-11 Supplemental Information 11 G3bp2 expression in sertoli cell GAPDH.

10.7717/peerj.13532/supp-12 Supplemental Information 12 PLZF numbers of per tubule.

10.7717/peerj.13532/supp-13 Supplemental Information 13 Sperm concentration.

10.7717/peerj.13532/supp-14 Supplemental Information 14 G3bp2 cKO WT SKO DKO.

10.7717/peerj.13532/supp-15 Supplemental Information 15 Stress granules containing germ cells of per tubule.

10.7717/peerj.13532/supp-16 Supplemental Information 16 G3bp2 cKO actin.

## Additional Information and Declarations

### Data Availability

The following information was supplied regarding data availability: The raw measurements are available in the Supplemental Files.
