# Supplementary figures and images for: G3BP2, a stress granule assembly factor, is dispensable for spermatogenesis in mice
Source: PeerJ. 2022 Jun 28;10:e13532. doi: 10.7717/peerj.13532 (PMC9248785; doi:10.7717/peerj.13532)

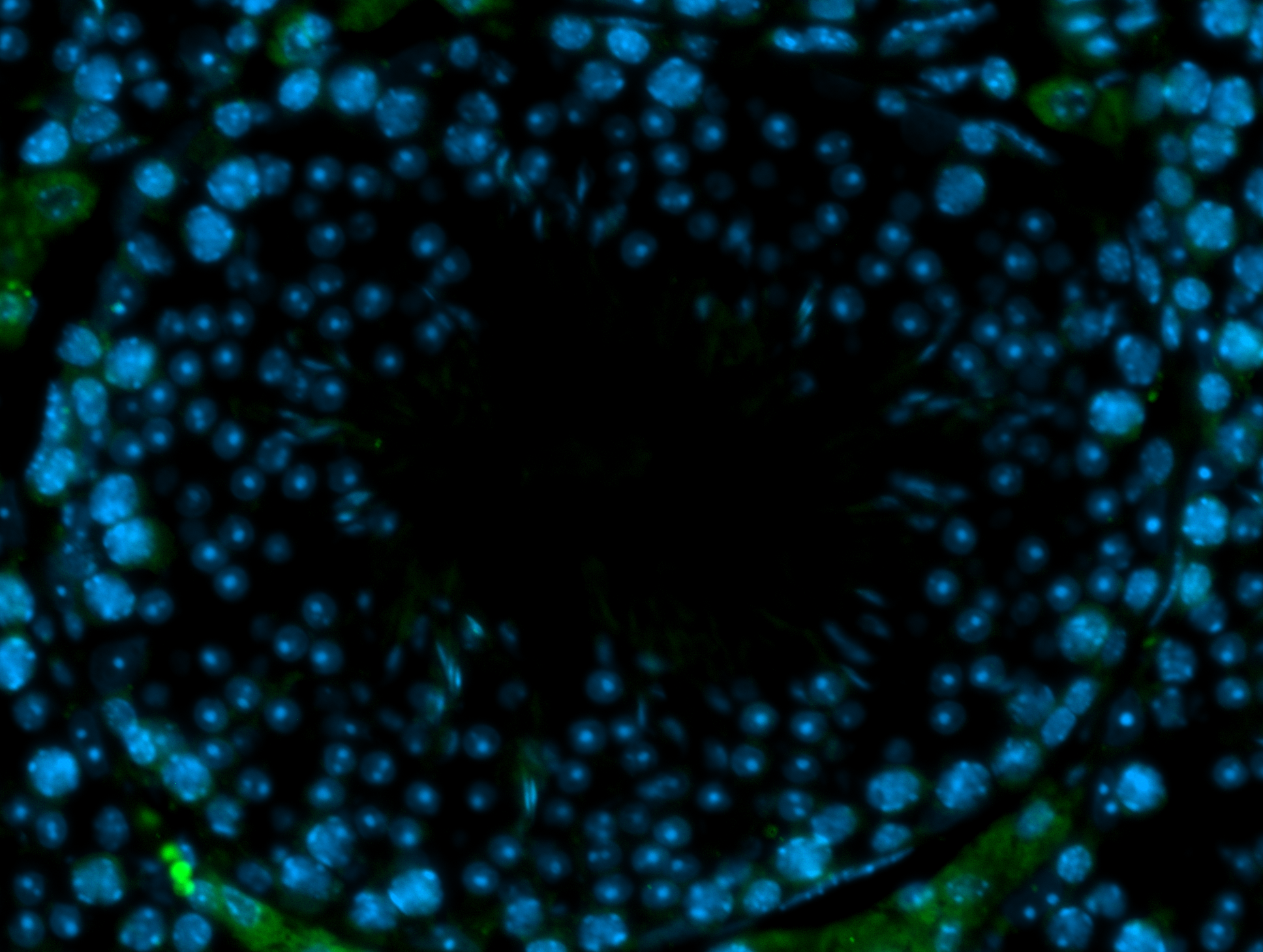

Supplement: Supplemental Information 6 [file peerj-10-13532-s006.tif]

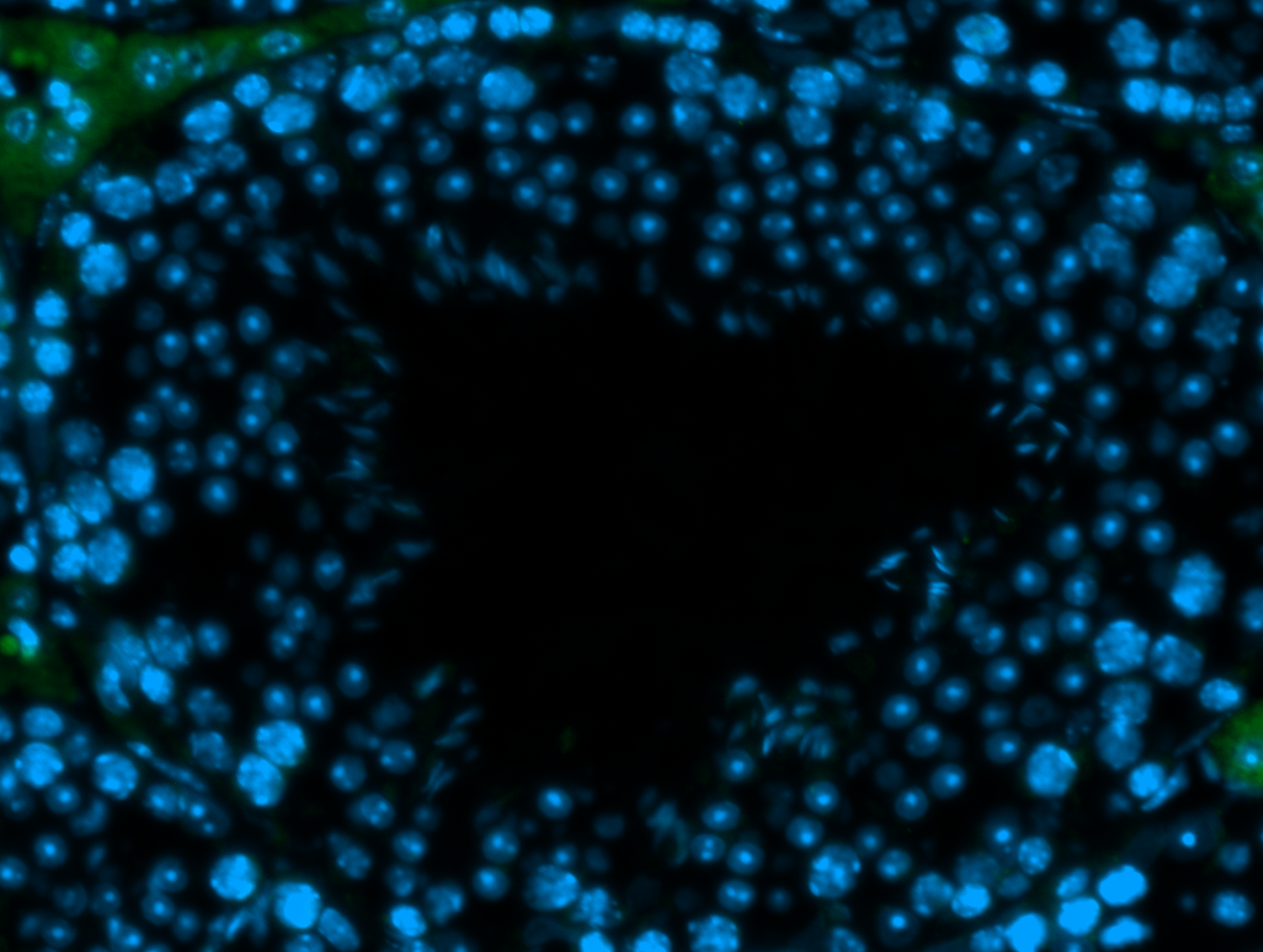

Supplement: Supplemental Information 8 [file peerj-10-13532-s008.tif]

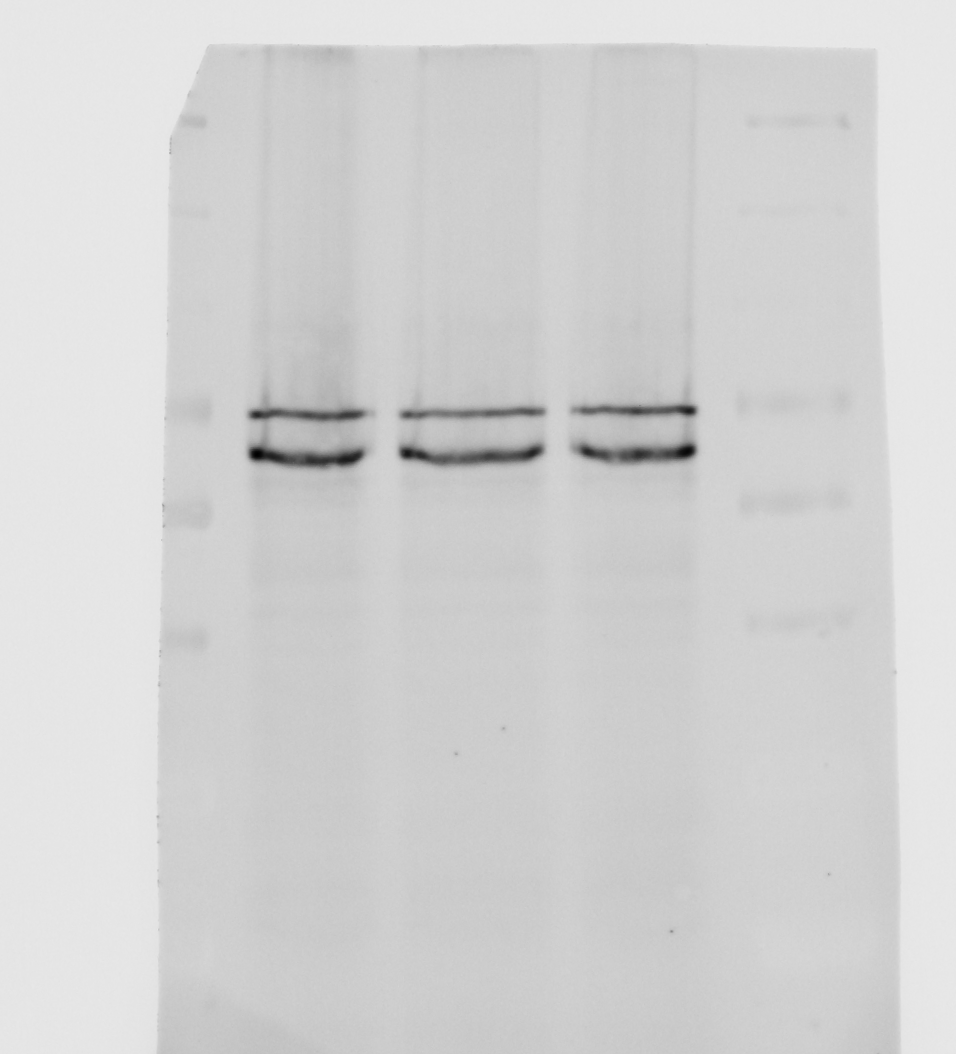

Supplement: Supplemental Information 9 [file peerj-10-13532-s009.tif]

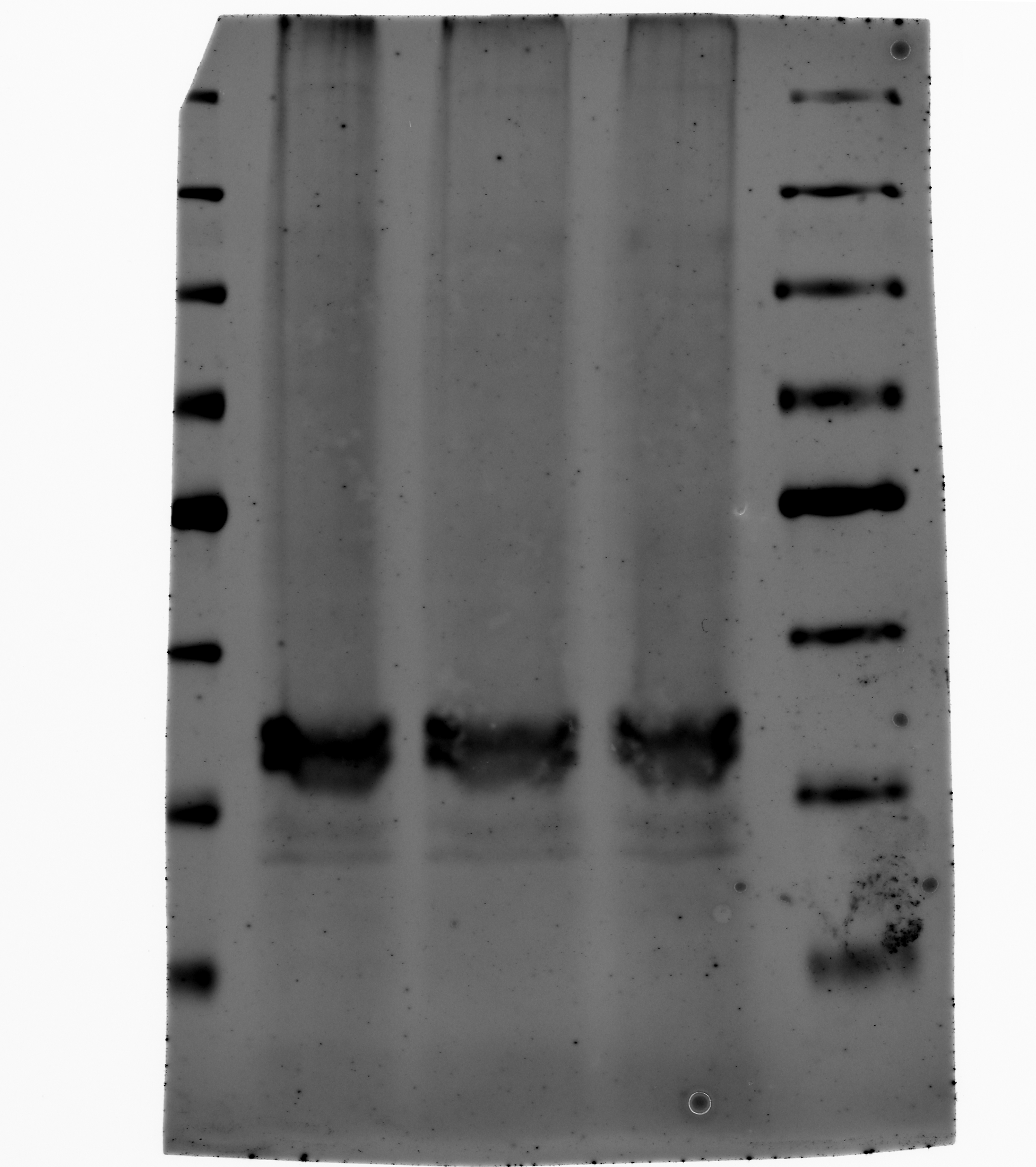

Supplement: Supplemental Information 11 [file peerj-10-13532-s011.tif]

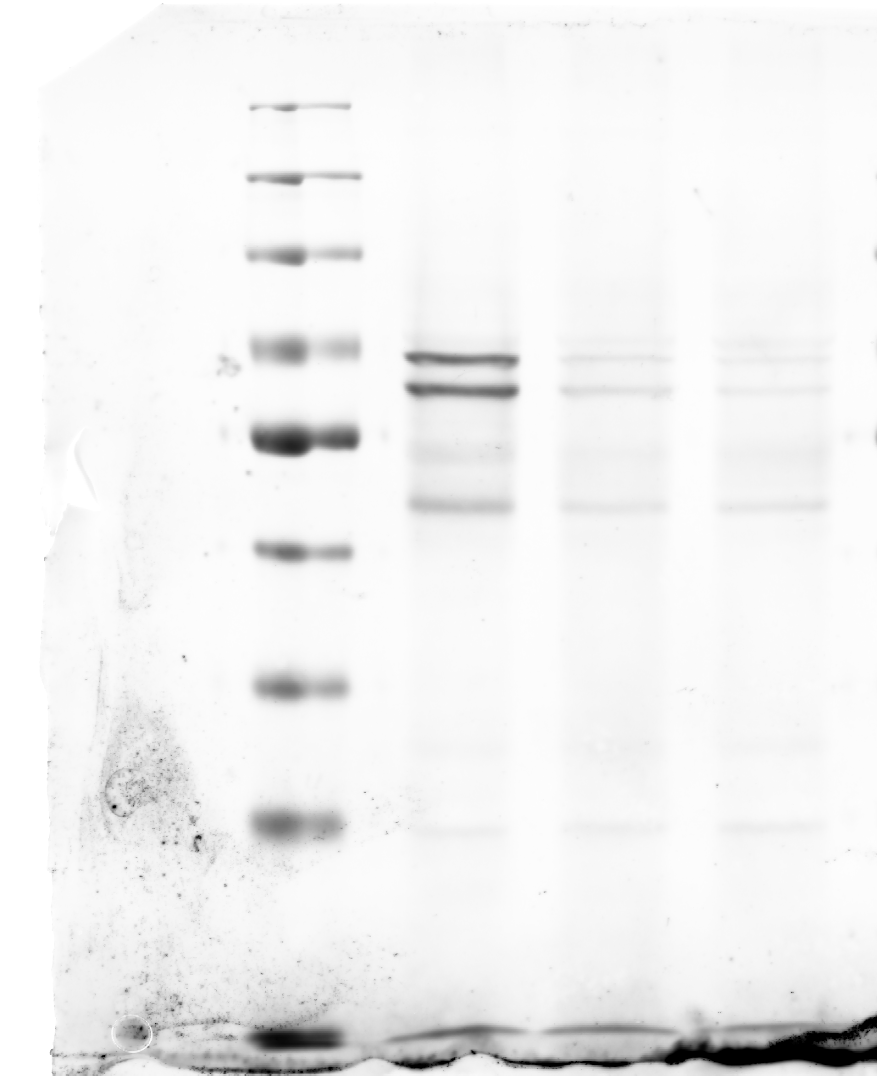

Supplement: Supplemental Information 14 [file peerj-10-13532-s014.png]

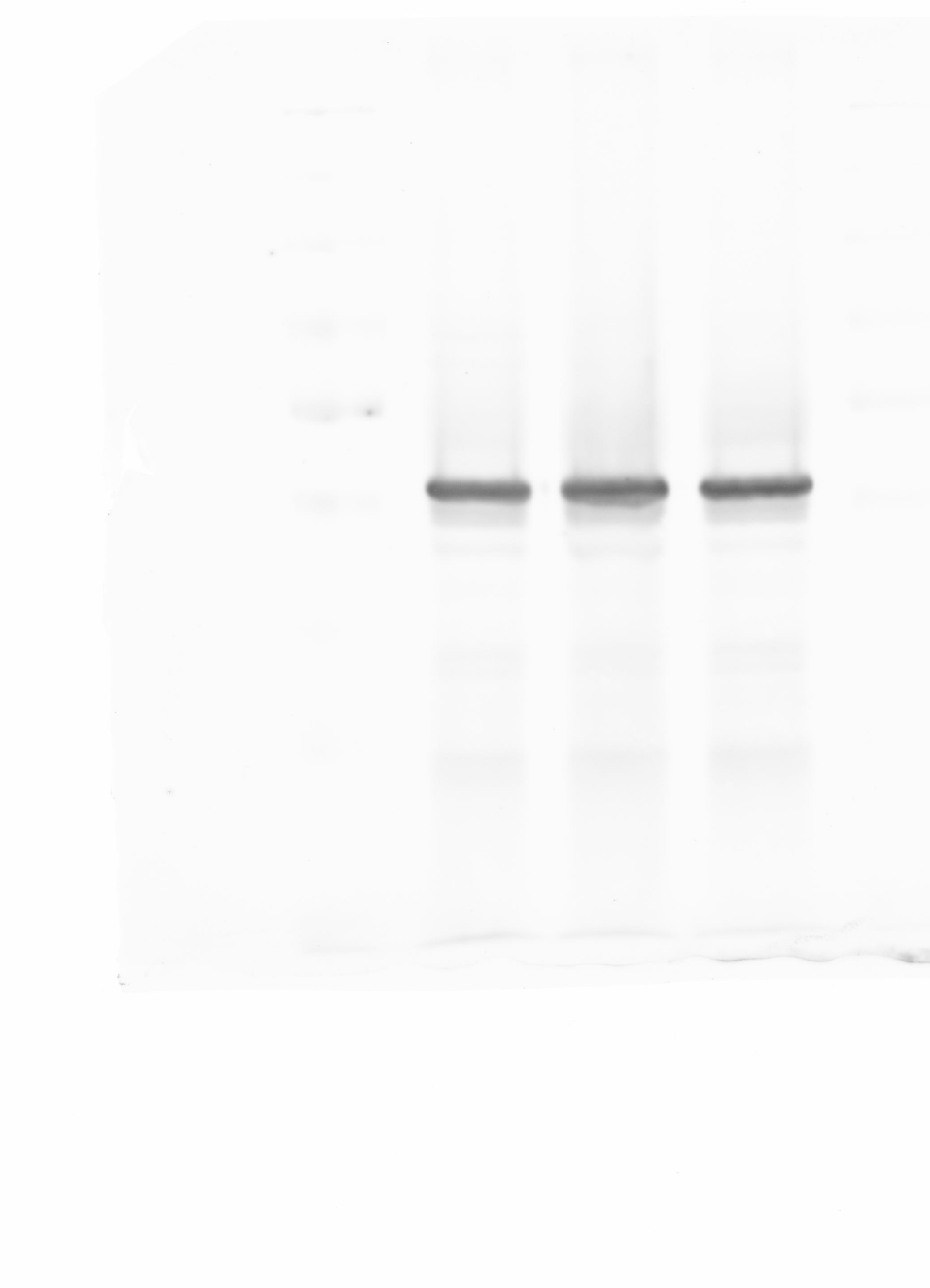

Supplement: Supplemental Information 16 [file peerj-10-13532-s016.png]
